# Visualization and Quantitative Evaluation of Functional Structures of Soybean Root Nodules via Synchrotron X-ray Imaging

**DOI:** 10.34133/plantphenomics.0203

**Published:** 2024-07-17

**Authors:** Alireza Nakhforoosh, Emil Hallin, Chithra Karunakaran, Malgorzata Korbas, Jarvis Stobbs, Leon Kochian

**Affiliations:** ^1^ Global Institute for Food Security, Saskatoon, SK S7N 4L8, Canada.; ^2^ Canadian Light Source Inc., Saskatoon, SK S7N 2V3, Canada.

## Abstract

The efficiency of N_2_-fixation in legume–rhizobia symbiosis is a function of root nodule activity. Nodules consist of 2 functionally important tissues: (a) a central infected zone (CIZ), colonized by rhizobia bacteria, which serves as the site of N_2_-fixation, and (b) vascular bundles (VBs), serving as conduits for the transport of water, nutrients, and fixed nitrogen compounds between the nodules and plant. A quantitative evaluation of these tissues is essential to unravel their functional importance in N_2_-fixation. Employing synchrotron-based x-ray microcomputed tomography (SR-μCT) at submicron resolutions, we obtained high-quality tomograms of fresh soybean root nodules in a non-invasive manner. A semi-automated segmentation algorithm was employed to generate 3-dimensional (3D) models of the internal root nodule structure of the CIZ and VBs, and their volumes were quantified based on the reconstructed 3D structures. Furthermore, synchrotron x-ray fluorescence imaging revealed a distinctive localization of Fe within CIZ tissue and Zn within VBs, allowing for their visualization in 2 dimensions. This study represents a pioneer application of the SR-μCT technique for volumetric quantification of CIZ and VB tissues in fresh, intact soybean root nodules. The proposed methods enable the exploitation of root nodule’s anatomical features as novel traits in breeding, aiming to enhance N_2_-fixation through improved root nodule activity.

## Introduction

Nitrogen (N) plays a pivotal role as a mineral nutrient in the growth and development of plants, serving as a fundamental constituent of essential biomolecules such as proteins, nucleic acids, and chlorophyll [1]. The modern agricultural system relies heavily on synthetic nitrogen fertilizers, without which it is projected that only half of the global population could be supported [2]. However, the production and application of nitrogen fertilizers entails substantial consumption of natural gas and fossil fuels, using about 1.5% of the world’s oil each year [3] for N fertilizer synthesis from N_2_ gas, and is the primary factor in agriculture’s considerable carbon footprint. Also, N fertilizer use has considerable negative environmental impact, resulting in soil greenhouse gas emissions (mostly N_2_O) and the pollution of ground and surface water sources by nitrates not absorbed by the plant roots [4,5].

Legume–rhizobia symbiosis, the most efficient N_2_-fixing system in plants, has long been recognized as a sustainable alternative to the use of N fertilizers. The process of the symbiotic nitrogen fixation (SNF) takes place in legume root nodules, specialized structures where N_2_-fixing rhizobia bacteria reside [6].

Nitrogen fixation is regulated by the plant’s nitrogen demand, the availability of nitrogen to the plant, and the amount of carbon the plant provides to the nodule. Research indicates that the rapid sequestration of fixed nitrogen and efficient water cycling between the shoot and nodules are of prime importance in promoting nodule activity [7]. The functional activity of nodules in transporting water and carbon (C) to the nodule and fixed N from the nodule to the plant is influenced by the anatomical features of the nodules [8,9].

Studies on the relationship between plant structure and function have provided evidence for the potential benefits of exploiting anatomical features of different plant organs for crop improvement, especially under suboptimal conditions. Various anatomical traits in plants, such as the number and size of the metaxylem in wheat [10] and soybean [11], the thickness of major veins in rice [12], and the formation of root cortical aerenchyma in maize [13,14], have been shown to confer tolerance to water and nutrient stresses and enhance crop productivity under unfavorable or low-input conditions. These findings suggest that a deeper understanding of the anatomical basis of plant function can lead to the development of crop varieties that use resources more efficiently.

Nodules are composed of 2 functionally important tissues: the central infected zone (CIZ), where rhizobia bacteria colonize and perform nitrogen fixation, and vascular bundles (VBs) that serve as conduits for the transport of water, nutrients (primarily carbon as photosynthetically derived sugars), and fixed nitrogen compounds between the nodule and the plant.

Despite the clear role of the symbiotic relationship between the legume plant and rhizobial bacteria, the functional importance of the nodular CIZ and VB tissues in nitrogen input to the plant, and nodule activity, remain poorly understood [8,9]. A quantitative assessment of these tissues is imperative to determine their functional importance in N_2_-fixation and to gain a better understanding of the physiological basis underlying the observed differences between high and low nitrogen-fixing genotypes of legume crops such as soybean.

Previous histological studies, utilizing light and electron microscopy, have provided a detailed anatomical description of the root nodule’s internal structures [15]. In soybean nodules, the CIZ is identified by its remarkable size in the center of root nodules. The CIZ is enclosed by a narrow band of non-infected parenchyma cells within which VBs are embedded [15,16]. Although these microscopy techniques produce high-quality images at high spatial resolution, they are largely restricted to 2-dimensional (2D) imaging, thereby limiting our ability to fully appreciate the intrinsic 3-dimensional (3D) architecture of CIZ and VB structures. To date, only 2 studies have provided a 3D representation of CIZ and VB structures in soybean root nodules using light [17] or x-ray microscopy [18] techniques. Although both studies contributed markedly to knowledge advancement, their methodologies required extensive, labor-intensive sample preparation and the use of contrast-enhancing agents.

In the last 2 decades, the synchrotron x-ray microcomputed tomography (SR-μCT) technique has emerged as a powerful tool in plant sciences, as reviewed by Indore et al. [19]. This non-destructive imaging technique allows for rapid, high-resolution visualization of internal structures of various plant organs [20–22] with minimal sample preparation. The penetrating power and short wavelength of illuminating x-rays enables thick specimens to be imaged at high spatial resolution without the need for thin sectioning [23]. The resulting image contrast is driven by differential x-ray attenuation due to the variation in tissue composition and density (e.g., dense lipid-rich structures versus water-rich cellular tissues [24]), allowing for tissue differentiation without staining procedures.

Synchrotron x-ray fluorescence (SR-XRF) imaging is another technique commonly used in plant sciences to provide in situ information on the distributions and concentrations of elements within the plant at different levels of spatial resolution, ranging from the whole plant to cellular organelles [25].

Iron (Fe), which plays an essential role in SNF, is predominantly localized in the CIZ tissue of nodules [26]. Fe functions as a cofactor for the key enzymes involved in N fixation, including nitrogenase, which catalyzes the reduction of N_2_ to NH_4_, and ferredoxin, which acts as an electron donor for nitrogenase. Additionally, Fe is required for the proper functioning of leghemoglobin, a protein that maintains a steady supply of low levels of oxygen in the microaerobic environment in which the reaction occurs, i.e., in the CIZ tissue of nodule [27]. At maturity, soybean nodules contain the highest concentration of iron in the plant, with 44% of the total plant iron present in the infected cells of nodules [28].

Zinc (Zn) is an essential micronutrient necessary for plant growth, as it performs key functions in numerous metabolic pathways. Nonetheless, the physiological range of tissue [Zn] from Zn deficiency to Zn toxicity is relatively narrow. Hence, the plant must protect against possible Zn toxicity [29]. To ensure survival by providing sufficient levels of essential Zn while preventing excess Zn accumulation resulting in Zn toxicity, plants require a well-regulated Zn homeostatic network encompassing import, trafficking, sequestration, and export processes [30]. For example, sequestering Zn in the root is one adaptive strategy employed by plants to maintain non-toxic levels of Zn in above-ground tissues [29]. The root endodermis, the innermost cortical layer surrounding the vascular cylinder (stele), plays a pivotal role in the sequestration of Zn within plant roots. Previous studies utilizing the XRF imaging technique have reported the predominant localization of Zn within the root endodermis in various crops, including soybean [31–33].

In light of these findings reporting the specific localization of Fe in the CIZ tissue of the nodule, as well as Zn sequestration in the plant root vasculature, and considering the continuity of plant root and nodule vasculature [17], this study speculated on the feasibility of non-invasively visualizing the CIZ and VB tissues in nodules by 2D mapping of Fe and Zn distributions in root nodules using SR-XRF.

In the present study, we employed high-resolution SR-μCT and SR-XRF imaging techniques for rapid and non-invasive visualization of functional structures in fresh, intact root nodules of soybean genotypes with varying N_2_-fixation efficiencies, in both 2 and 3 dimensions. Additionally, we present here the successful application of Biomedisa, an open-source online platform recently developed for semi-automated segmentation of volumetric images [34], to successfully and rapidly segment nodular CIZ and VB tissues in SR-μCT image data, thereby speeding up the quantitative assessment process. The SR-μCT and SR-XRF imaging techniques allow for quick imaging of multiple nodules at once, making it possible to employ them for high-throughput phenotyping of internal structure of root nodules in their natural hydrated state, providing novel information that might be used to identify genotypes with more active N-fixing root nodules.

## Materials and Methods

### Plant material and experimental conditions

This study utilized 2 different sets of soybean genotypes for each experiment conducted at the BMIT-BM and BioXAS-Imaging beamlines at the Canadian Light Source (CLS), Saskatoon, Canada. Each experiment was performed using 3 genotypes that varied in their N_2_-fixation capacity and root system size (Tables S1 to S3). The N_2_-fixation efficiency of the genotypes was assessed through ^15^N natural abundance analysis to estimate the percentage of plant N derived from the atmosphere, %Ndfa [35].

The soybean genotypes Williams 82, PI567651, and PI209332 were used for 3D visualization and volume quantification of the nodule structures, CIZ and VB, through SR-μCT imaging at the BMIT-BM beamline. At the BioXAS beamline, the soybean genotypes Dundas, Woodstock, and Gaillard were used to visualize the functional structures of soybean root nodules through XRF imaging. These soybean genotypes were used because they are short-season, adapted to growth in the western Canadian and US prairies, and were selected through the phenotyping of a set of 25 Canadian short-season soybean genotypes for traits associated with N_2_-fixation.

For experiments at both beamlines, soybean plants were grown under controlled conditions at day/night temperatures of 28 °C/20 °C, with a 16-h/8-h photoperiod, light intensity at a plant height of 350 µmol m^−2^ s^−1^, and 50% relative humidity, in a growth chamber at the Global Institute for Food Security in Saskatoon, Canada. To induce nodulation, 4-day-old soybean seedlings, initially germinated and grown on a rolled germination paper and suspended vertically in water, were inoculated with *Bradyrhizobium japonicum* sourced from Novozymes NexusBioAg (Cell-Tech liquid). The seedlings were then transplanted into Sunshine Mix #2 with low N content from Sun Gro Horticulture. Throughout the 4-week period of plant growth, the plants were watered twice a week with 1/3 strength of the nitrogen-free nutrient solution as was done in McClure and Israel [36]. Prior to imaging at the synchrotron, the nodulated roots of soybean plants were carefully washed in water to remove the attached soil, and the intact nodules were gently collected.

### Experimental setups at the BMIT-BM beamline

#### Data collection and tomographic reconstruction

Tomographic scans were collected at the BMIT-BM beamline (05B1-1) of the CLS (https://bmit.lightsource.ca/about/Introduction/). In this experiment, the white beam was attenuated by a 0.1-mm-thick silver filter to reduce high radiation absorption resulting from low-energy x-rays, which can lead to damage to the sample, and to generate a mean beam energy of 25.5 keV. Projections were collected by a PCO Edge 5.5 (2,560 × 2,160 pixels) sCMOS detector that was coupled to a 10-μm-thick LSO:Tb scintillator (European Synchrotron Radiation Facility) by means of an optical system (Optique Peter, Mitutoyo LWD Plan Apochromat) with 10× magnification. This setup resulted in an effective pixel size of 0.72 μm and a field of view of 1.85 (H) mm× 1.56 (V) mm.

The fresh and intact single medium-sized (~1.5 mm diameter) root nodules were placed inside 1.5-ml microcentrifuge tubes and secured in place using a Kim wipe (Kimtech) to prevent nodule movement during the scan. The tubes, bearing the sample, were affixed on a Huber manual goniometer head using dental wax and then mounted on the rotation stage. The distance between the sample and the detector was set to 4.0 cm. To correct x-ray images for a non-homogeneous beam profile and normalize the intensity, 20 flat-field (no sample) and 20 dark-field (no x-rays) images were acquired. For each sample, 1,800 projection images were acquired over 180° of sample rotation, with an exposure time of 30 ms for each projection image. Therefore, a complete SR-μCT scan of a single nodule took ~1 min.

Two-dimensional projection images were reconstructed to generate 3D tomographic volumes via use of a filtered back projection algorithm implemented in the UFO-KIT software (https://github.com/ufo-kit). We used EZ-UFO (https://github.com/sgasilov/ez_ufo) that provides a graphical interface to the data reconstruction tools of the UFO-KIT software [37–39]. Image processing included removal of large spots that stem from defects in the scintillator crystal, flat- and dark-field correction, phase retrieval via the transport of intensity approach, and suppression of ring artifacts. Before the final reconstruction, a test image stack was generated in UFO to find the optimized values for the reconstruction parameters. The ring artifacts were suppressed using the Sarepy sorting algorithm. For phase-retrieval, a Paganin filter module [40] was employed with an x-ray energy of 25.5 keV, an effective pixel size of 0.72 μm, a propagation distance (sample to detector) of 4 cm, and a δ/β ratio of 100. The histogram clipping values for converting 32-bit TIFF image stacks to 16-bit TIFF image stacks in the final reconstructions were determined using the test image stack. The Avizo 3D 2021.1 (Thermo Fisher Scientific) imaging software was used for 3D visualization and volume renderings of the final image stacks and to produce the videos found in the Supplementary Materials.

#### Segmentation and volumetric quantifications of nodule CIZ and VB tissues

Following the volume reconstruction of root nodules, we used Biomedisa, an open-source online platform developed for semi-automatic segmentation of volumetric images, to obtain 3D models of internal structures of the root nodule, i.e., CIZ and VBs (Fig. S1). Biomedisa utilizes both the labeled image and the original image stacks as input data. The labeled image consists of sparsely pre-segmented slices, which serve as reference slices. By employing a smart interpolation algorithm, Biomedisa assigns labels to the features of interest within the unlabeled slices located between the pre-segmented slices in the tomographic volume [34].

Before manual segmentation, the original image stack was converted from 16-bit to 8-bit images and subsampled by a factor of 2. This step was employed to decrease the size of the pre-segmented image data and hence reduce the computation time required for segmentation of the remaining unlabeled slices by Biomedisa. The CIZ and VB structures were manually labeled on multiple slices using the Avizo’s Segmentation Editor and saved as separate labeled images. For the CIZ, labels were assigned manually every 100th slice. However, due to the complex architecture of the nodule vasculature, a denser labeled image was required to achieve high segmentation accuracy and minimize interpolation errors by Biomedisa. Therefore, manual segmentation of the VB tissues within the nodule was performed on every 40th slice. On average, the label images for CIZ and VBs consisted of 25 and 10 reference slices, respectively. Manual segmentations were carried out using the freehand mode of the lasso tool, with the auto-trace option being active, which facilitate the segmentation process through auto-tracing of the edges of the structures.

Following the manual segmentation, the labeled and original image stacks were exported to Biomedisa. The (semi) automated segmentation process utilized the default configurations in Biomedisa. The interpolation of the labels to generate a fully segmented volume (3D model) took approximately 10 min of computation time.

Following the completion of the segmentation by Biomedisa, the resultant 3D models of CIZ and VB structures were imported back in Avizo and checked visually for errors and artifacts. If any gaps or discontinuities were found in the fully labeled volumes, additional slices within the gap regions were manually labeled, and the corrected labeled images were exported back to Biomedisa to regenerate the 3D models (Fig. S1). Post-processing of the 3D models involved smoothing, filling holes, and removal of outliers (unconnected voxels or islands), which were performed in Avizo. Subsequently, volumetric quantifications were conducted using the Volume Fraction tool in Avizo. The relative volumes of the CIZ and VB tissues were determined by calculating the ratio of their volumes to the total volume of the nodule. The total volume of the nodule was obtained using a similar segmentation approach as described for CIZ.

### Experimental setups at the BioXAS-imaging beamline

#### Data collection and analysis

The SR-XRF imaging data were collected at the BioXAS-Imaging undulator beamline of the CLS (https://bioxas-imaging.lightsource.ca/) equipped with a double-crystal Si(111) monochromator, an upstream vertically collimating, harmonic-rejecting mirror with a rhodium (Rh)-stripe, and a downstream vertically and horizontally focusing Rh-coated mirror. The main optics creates a focused secondary source (SS) in the experimental hutch providing the light for the 2 distinct spatial resolution modes. In the macro mode, the beam size on samples is varied by using circular W apertures positioned downstream of the SS. In the micro mode, the SS is demagnified to either 5 or 2 μm by a set of Rh-coated Kirkpartrick–Baez mirrors.

The SR-XRF imaging data acquisition was performed in the macro mode on fresh, intact root nodules (Fig. S2). For this experiment, a total of 15 individual root nodules were collected, with 3 plants for each genotype contributing 5 nodules each. We deliberately chose the mature root nodules to ensure their optimal elemental contents.

To minimize dehydration and prevent any sample movement during the scanning of nodules, a set of 5 individual nodules were carefully placed between 2 Kapton films. The nodules were arranged in a row, with an approximate 1-cm space maintained between adjacent nodules. The backing Kapton layer was adhesive and thicker (25.4 µm, Kapton Tape) compared to the non-adhesive Kapton film (7.6 µm, Kapton Thin-Film) facing up toward the incident beam. The 4-element silicon drift Vortex-ME4 detector was set at a 45° angle, while the samples were positioned in a 90° stage configuration relative to the incident x-ray beam. This configuration minimizes the artifacts in the images related to sample thickness. The SR-XRF spectra were collected at room temperature in continuous bi-directional fly-scanning mode. The beam energy was set to 15 keV. The spatial resolution was set at 20 μm and a dwell time of 20 ms. On average, it took 30 min to complete a full scan of an individual nodule with a scanning area of 5 mm × 5 mm including the overhead time. We observed no signs of beam-induced damage or changes in shape caused by dehydration of nodules after the scans.

To perform fine mapping of Fe and Zn distributions within the root nodule, we used the BioXAS-Imaging micro mode (5 μm and 2 μm beam size) on root nodule sections (Fig. S2). The silicon drift Vortex-ME3 detector, consisting of 3 elements, was set at a 90° angle, while the samples were positioned in a 45° stage configuration relative to the incoming x-ray beam. Hydrated nodules, embedded in 5% agarose, were used to obtain 100-μm-thick sections using a vibratome. Each section was individually sandwiched between 2 Kapton films, as described above for the preparation of intact root nodules for XRF imaging. The fine mapping of Fe and Zn distributions was performed at a resolution of 5 μm for the entire nodule section, and at 2 μm within a predefined region of interest (ROI) containing 3 VBs. The sections dedicated to elemental fine mapping within the region containing VB tissues underwent an initial fast, low-resolution scan (30 μm spatial resolution with a dwell time of 20 ms), and the positions of the VBs within the nodule section were identified through mapping Zn on-the-fly. Once the ROIs were identified on the nodule section, 4 ROIs were defined and scanned at a resolution of 2 μm with an exposure time of 100 ms. For the whole section scans at 5 μm, cv. Woodstock’s nodule section was used, while for the elemental fine mapping within the VBs regions, the nodule section was obtained from cv. Dundas.

The acquired spectra were processed using PyMca 5.8.7 software [41], which included peak fitting and the generation of elemental maps. The elemental distribution maps were obtained for Fe, Zn, and other elements that are within the energy window between K and Zn and are known to be associated with N_2_-fixation such as Co, Ni, and Cu [42]. After spectral deconvolution, the estimation of Fe abundance in root nodules was conducted using a semi-quantitative approach that involved counting the x-ray photons emitted from the samples and normalizing them to the incident beam. To quantify Fe, the total XRF counts under the Fe peak were calculated by PyMca. However, we note that the abundance of Fe within the nodules was not corrected for self-absorption resulting from the variations in the nodules thickness across the nodule geometry.

#### Statistical analysis

The analysis of variance (ANOVA) was performed for traits related to N_2_-fixation (Tables S1 to S3) and the abundance of Fe in root nodules, determined by XRF counts. The Proc MIXED procedure in SAS 9.2 (SAS Institute, Inc., Cary, NC) was employed for this analysis. To assess the statistical significance of mean differences, the least significant difference (LSD) test was utilized at a critical significance level of *P* = 0.05.

## Results and Discussion

### Three-dimensional visualization and quantitative analysis of functional structures of soybean root nodules using SR-μCT

**Fig. 1.**
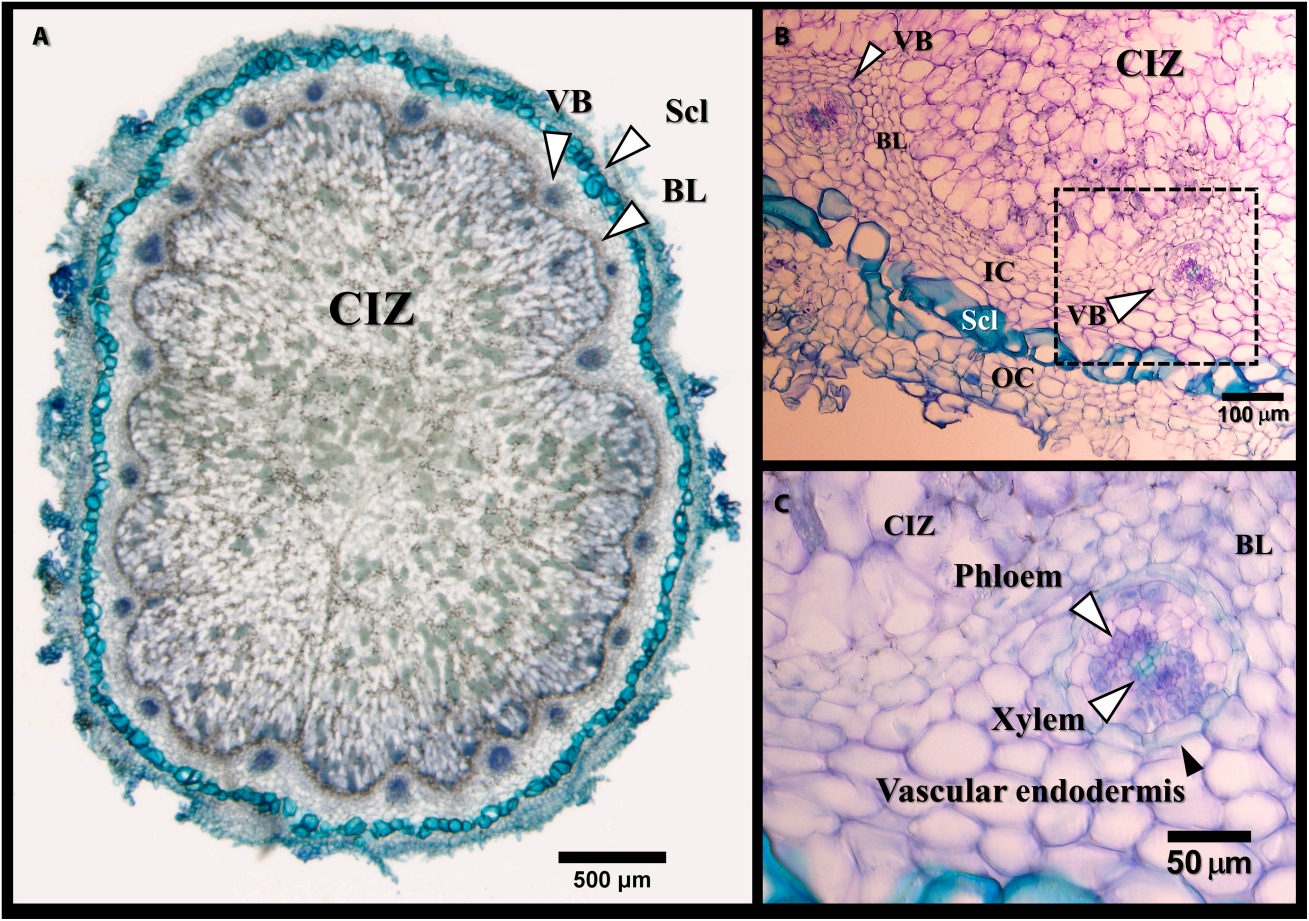
Light micrographs of toluidine blue-stained transverse sections of a soybean nodule (cv. Dundas). The sections, 50 μm thick (A) and 20 μm thick (B and C), were obtained from fresh nodules embedded in 5% agarose. The micrographs depict the anatomical features of the nodule, including bacteroid-containing cells of the central infected zone (CIZ), the inner cortex (IC) and outer cortex (OC), boundary layer (BL) cells surrounding the infected zone, scleroid layer (Scl) cells, vascular bundles (VB), the vascular endodermis, and xylem and phloem cells. Micrograph B was taken in the cortex region where 2 VBs are present. The peripheral layer of the sclerenchyma cells (in both A and B) is distinguishable by its cells’ size and thick walls, which are blue-green after staining with toluidine blue. The CIZ, located in the center of the nodule, is easily identifiable by the densely packed non-infected parenchyma cells of the BL surrounding it (A to C). Micrograph C represents an enlarged area, identified by a black box within micrograph B, focusing on a region within the inner cortex consisting of an individual VB. Within the VB, the arrowheads indicate the presence of a vascular endodermis (black arrowhead) surrounding the VB, and xylem cells and phloem cells (white arrowheads). The xylem vessels at the center of the VB are identified by their distinct green color, while the adjacent phloem cells are purple in color. The nodule section was obtained from a 28-day-old soybean plant (cv. Dundas), inoculated on day 4.

Root nodules consist of several types of tissues, of which CIZ and VBs play important roles in N_2_-fixation. The light micrographs in Fig. 1 represent the different tissues of a soybean root nodule. The peripheral layer of sclerenchyma cells, separating the inner and outer cortices, is distinctively visible due to the thickness of their walls and the relatively larger size of their cells (Fig. 1A and B). In a mature soybean root nodule, the CIZ tissue occupies a very large fraction of the nodule and is surrounded by the inner cortex, which is a narrow band of non-infected parenchyma cells, within which VBs are embedded [15]. Similar to plant roots, nodule VBs consist of xylem, phloem, pericycle, and vascular endodermis, which is composed of densely packed cells, enveloping all elements of the VB (Fig. 1C) [43].

The 3D visualization and quantitative analysis of nodular CIZ and VB tissues were assessed across 3 soybean genotypes with varying N_2_-fixation efficiencies as previously determined through the destructive method of ^15^N natural abundance. Based on the results obtained from phenotyping these genotypes for traits related to N_2_-fixation (Table S1), genotypes Williams 82 and PI567651 exhibited significantly higher N_2_-fixation compared to PI209332. Williams 82 was characterized by its significantly larger root system, while PI209332 had the smallest root system.

The SR-μCT at submicron pixel resolution was employed to acquire high-quality tomograms of fresh soybean root nodules in a non-invasive manner. These tomograms were then volume rendered into a 3D representation, as shown in Movie S1. Synchrotron imaging using the SR-μCT technique provides a high photon flux that enables rapid acquisition times and high-speed imaging of root nodules (less than 1 min for complete scanning of one nodule). This capability enables the preservation of the structural integrity of root nodules without any perceptible damage after the scanning process.

**Fig. 2.**
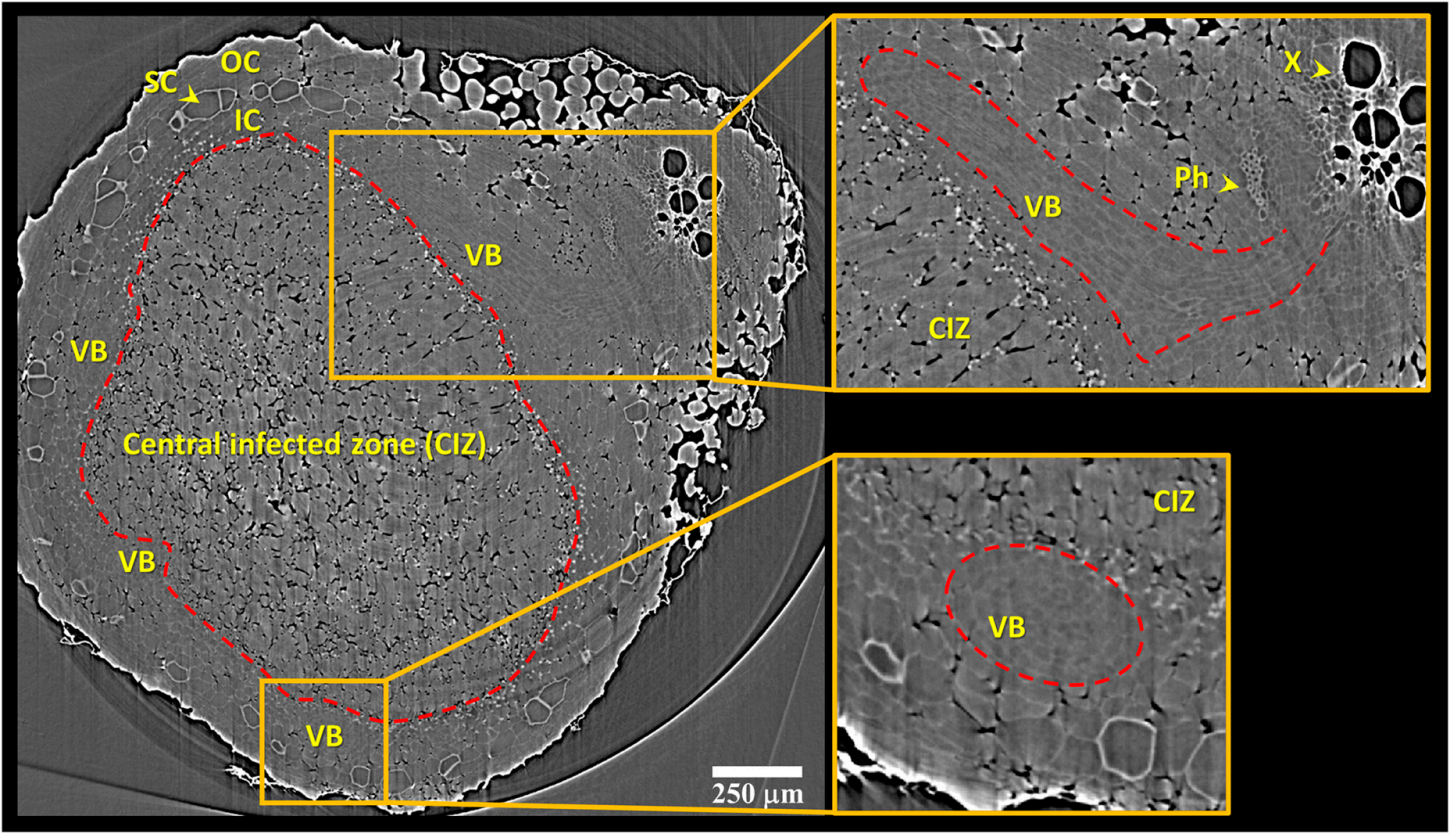
Synchrotron x-ray micro-tomogram of a fresh root nodule of a 4-week-old soybean plant (genotype PI209332). The water-filled cells from various tissues within the root and nodule appear gray, while air-filled intercellular spaces, particularly noticeable in the large embolized xylem vessels (X) within the root, appear black, due to their lower x-ray attenuation. The central infected zone (CIZ), outlined in red, is readily identified by the tightly packed non-infected parenchyma cells of the boundary layer that surrounds it. Magnified portions of the tomographic nodule section are shown in inset boxes, with the VB’s outlines highlighted in red. The scleroid layer (SC), separating the inner (IC) and outer (OC) cortices, is labeled.

Figure 2 shows a transverse micro-tomogram of a fresh nodule attached to the root with the main cellular tissues labeled in both specific cell types and tissues in the nodule and root (e.g., X [for xylem] and Ph [for phloem], in the root). The spatial resolution of 0.72 μm used in this study was sufficient to visually dissect the CIZ and VB tissues within the nodule tomograms. The individual cells within the nodule VBs could also be resolved. The sclerenchyma cells are also clearly detectable due to their thick walls and comparatively higher x-ray attenuation. As seen in the Fig. 1 light micrograph, the CIZ tissues is surrounded by the boundary layer, which is the innermost cellular layer of the inner cortex. The boundary layer is characterized by the absence of intercellular spaces (Fig. 1A to C). This characteristic facilitates the differentiation of the CIZ tissue on the nodule micro-tomograms (Fig. 2). The CIZ tissue is composed of relatively loose cells that can be readily differentiated from the surrounding nodule tissues based on the tightly appressed cells in the boundary layer. Similarly, the tightly packed layer of endodermal cells surrounding the nodule VBs facilitates the differentiation of VB tissues from the neighboring parenchyma cells of the nodule cortex (insets in Fig. 2, also cf. Fig. 1C). However, depending on the orientation of the VB within the nodule and the plane of volume slicing, the VBs appear in different forms in a tomographic section, as shown in the insets in Fig. 2.

The vascular connection between the plant root and nodule is transiently shown in Movie S2. The animation displays sequential *XY* plane slice of a 3D reconstructed nodule along the *Z*-axis from top of the root nodule downward. The movie is slowed at the root and nodule junction where nodule vascular strands at the tip of the red arrow appear in *XY*-specific slices and are protruding from the root vasculature in those specific *XY* plane slices. The connectivity and linkage between root and nodule vasculature become more evident when a relatively large nodule vessel, which is embolized and hence exhibits good contrast due to being air-filled, transiently appears as the movie proceeds through the nodule, with the nodule vessel protruding from the root stele into the nodule.

**Fig. 3.**
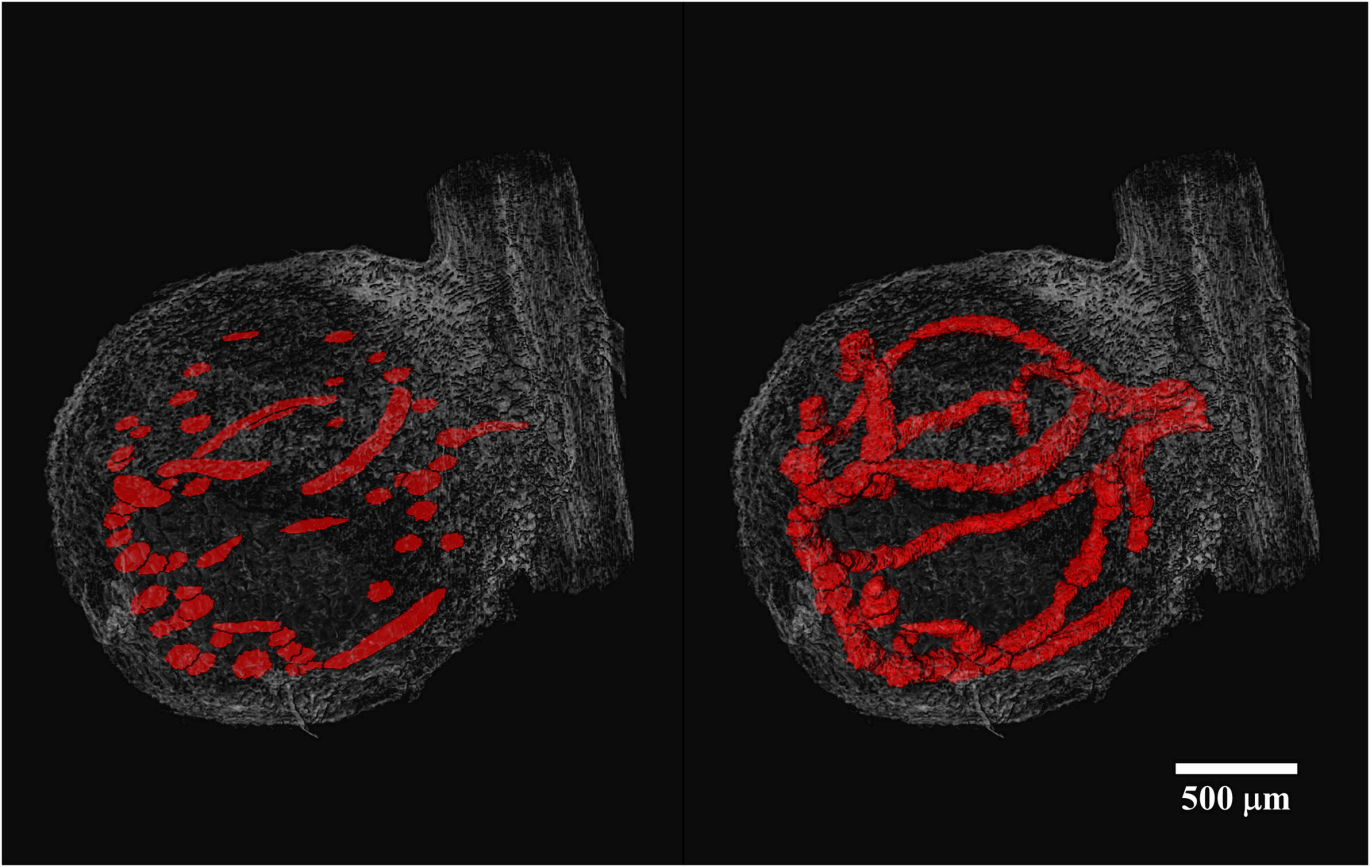
A representative 3D model of soybean nodule vasculature obtained through a (semi)automated segmentation approach using the smart interpolation algorithm implemented in Biomedisa. The manual segmentation of the VBs was performed on every 40th slice within the tomographic volume of the nodule (left panel). The Biomedisa’s interpolation algorithm assigned labels to the vascular tissues within the unlabeled slices located between the pre-segmented slices in the tomographic volume of the nodule, resulting in the reconstruction of a 3D model of nodule vasculature (right panel). The tomographic nodule volume was obtained through the reconstruction of synchrotron x-ray μ-CT images acquired from a fresh root nodule (genotype PI209332).

**Fig. 4.**
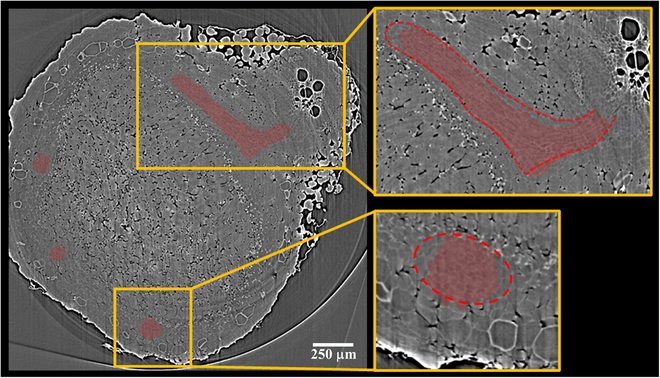
Visual assessment of the accuracy of the (semi)automatic segmentation of soybean nodule vasculature using Biomedisa’s smart interpolation algorithm. The image displays the Biomedisa’s segmentation results of the VBs on a slice located an equal distance from 2 manually pre-segmented slices. The insets highlight the Biomedisa’s performance in segmenting 2 VBs within the nodule tomogram. In contrast to the more accurate identification of the VB in the upper inset, the segmentation of the VB in the lower inset exhibits some partial loss of VB features that can be corrected if desired to improve the results.

Figure 3 depicts a representative 3D model of nodule vasculature (genotype PI209332). In contrast to the CIZ, the nodule vasculature exhibits a more complex structure, requiring denser reference labeled images for smart interpolation by Biomedisa to yield satisfactory results. The initial observations revealed that manual labeling every 40th slice and 100th slice in the tomographic volume is necessary to achieve satisfactory flawless and error-free 3D models of VBs and CIZ structures. Figure 4 shows the accuracy of Biomedisa’s smart interpolation algorithm in segmenting VB tissues within a nodule slice located equidistant from 2 manually pre-segmented slices. It should be noted that in our approach, we relied on visually assessing Biomedisa’s segmentation results and iteratively correcting errors and artifacts until achieving satisfactory segmentation outcomes. However, as described by Lösel et al. [34], the accuracy of Biomedisa’s segmentation results can also be quantitatively evaluated using metrics such as the Dice similarity coefficient (Dice) and the average surface distance (ASD).

**Fig. 5.**
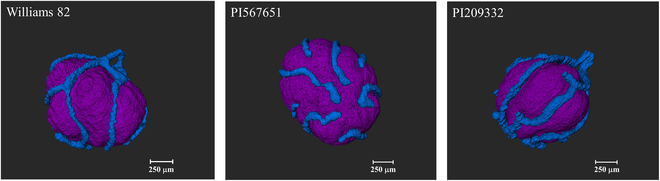
Three-dimensional view of the reconstructed nodule internal tissues of the central infected zone (CIZ, purple) and nodule vasculature (blue) in one nodule each from the 3 soybean genotypes we studied that vary in N_2_-fixation. Note the continuity of nodule vasculature surrounding the CIZ in the nodules of all 3 genotypes. The dual vascular connection between the nodule and root vasculatures was observed in all genotypes (here, solely visible from lateral and dorsal views, not frontal view in genotype PI567651).

The animation S3 in the Supplementary Materials displays a representative 3D rendering of the segmented structures of CIZ and VBs projected into the volume-rendered soybean root nodule. Figure 5 reveals that in all 3 soybean genotypes, the nodule vasculature forms a continuous network surrounding the entire infected zone. Also, the presence of a dual vascular connection between the nodule and root vasculature was evident in all genotypes (Fig. 5). These results closely resemble the previously reported 3D representation of CIZ and VBs structures in the soybean root nodule by Livingston et al. [17] using light microscopy.

The volumetric quantification of CIZ and VB structures in the 3 soybean genotypes that vary in N_2_-fixation revealed a greater genotypic variation in the volume of root nodule vasculature compared to the volume of CIZ (20.5% vs. 7.4%, Table). However, the relatively smaller variation observed between genotypes in the sizes of their CIZ volume could be attributed to the intentional selection of medium-sized nodules for this analysis (1.4 to 1.5 mm, Table). On average, CIZ occupies about 35% of the nodule volume (NOD), while only 1.7% of nodule volume is occupied by nodule vasculature. Williams 82 contrasted with PI567651 for VB/NOD volume ratio (1.2% vs. 2%).

**Table.** Volumetric quantification and volume fraction analysis of central infected zone and vascular tissues of root nodules in 3 soybean genotypes with varying N_2_-fixation

Genotype	Diameter (mm)	Central infected zone volume (CIZ) (mm^3^)	Vascular bundle volume (VB) (mm^3^)	Nodule volume (NOD) (mm^3^)	CIZ (% of the NOD volume)	VB (% of the NOD volume)
Williams 82	1.5	0.56	0.02	1.76	31.8	1.2
PI567651	1.4	0.59	0.03	1.58	37.4	2
PI209332	1.4	0.51	0.03	1.47	34.7	1.8
Mean ± SD	1.5 ± 0.04	0.55 ± 0.04	0.03 ± 0.005	1.60 ± 0.15	34.7 ± 2.80	1.7 ± 0.42
CV (%)	3	7.4	20.5	9.2	8.1	25.3

To date, the studies by Livingston et al. [17] and Duncan et al. [18] remain the only publications to document successful 3D reconstruction of internal CIZ and VB tissues in soybean nodules, employing light or laboratory-based x-ray microscopy. The first study by Livingston et al. [17] employed a laborious and destructive methodology that involved extensive sample preparation including fixation, dehydration, and embedding of the root nodules in paraffin, followed by sequential thin sectioning of the nodules. This approach required staining and then light microscopy imaging of more than 250 sections per nodule to enable the reconstruction of 3D models of nodular CIZ and VB structures from the 2D optical images of nodule sections. In the second study, Duncan et al. [18] employed x-ray microscopy and provided a highly detailed 3D representation of internal structures of soybean root nodules at cellular resolution. While their approach enabled non-invasive visualization of nodular CIZ and VB structures, achieving high-resolution, high-quality scans using a lab-based x-ray source required substantially prolonged scan durations. Scan durations ranged from 12 to 19 h per nodule, depending on the targeted resolution (2.2 vs. 1.1 μm, respectively). Furthermore, the long-duration, high-resolution scans necessitated a specialized sample preparation process, which involved fixing the nodules in a contrast enhancement agent for 35 days and embedding them in agarose. It is worth noting that neither of these studies provided quantitative insights into the nodular CIZ and VB tissues.

Our study demonstrated that, despite its limited accessibility and higher cost, synchrotron radiation’s brilliant, tunable, and high-resolution capabilities enabled the acquisition of high-quality images of root nodule’s internal structures with sufficient contrast. This distinguishes our study from the prior work by Livingston et al. [17] and Duncan et al. [18]. The primary novelty of the current study lies in its pioneering utilization of SR-μCT for the non-invasive and rapid imaging of functional structures of root nodules using fresh, intact root nodules, without the need for labor-intensive or specialized sample preparation. Additionally, this work introduces the first successful application of Biomedisa’s smart interpolation algorithm for the rapid segmentation (within ~10 min) of nodular CIZ and VB tissues on SR-μCT image data, speeding up the quantitative assessment process.

It should be noted that in our experiment, μCT scans were collected for only one nodule from each soybean genotype. This limitation hinders the ability to draw conclusions regarding the potential association between N_2_-fixation efficiencies of genotypes and the size of CIZ and VB structures in their nodules. The novelty of the present study, however, lies in its pioneering application of SR-μCT for non-invasive, rapid imaging of fresh, intact root nodules, without any sample preparation, to quantitatively assess soybean nodular CIZ and VB tissues. This innovative approach establishes SR-μCT as a powerful imaging tool for future studies targeting such structure–function assessments. Synchrotron imaging has proven to be a valuable imaging system for the non-invasive analysis of plant internal microstructures and to advance our understanding of structure–function relationships in plants. Kim and Lee [44] used synchrotron x-ray microscopy to study the role of xylem vessel anatomical characteristics in the recovery of embolized vessels and sap hydraulics in rice leaves. The study found that perforation plates play an important role in refilling embolized vessels and maintaining hydraulic efficiency. In another study, Matsushima et al. [21] employed SR-μCT and ascribed the variance in the longevity of rose varieties to dissimilarities in their VBs and peduncle pith structures. Cloetens et al. [45] used synchrotron x-ray phase tomography (SR-PCT) to visualize and quantify the 3D network of intercellular air spaces in mature Arabidopsis seeds. In dry seeds, limited seed coat permeability strongly impedes gas exchange, making the air space a potential storage space for oxygen needed during seed imbibition. The Cloetens et al. [45] study presented another example of the value of synchrotron radiation for non-invasive quantitative analysis of plant internal microstructures, which cannot be achieved using other methods.

### Two-dimensional visualization of functional structures of soybean root nodules using SR-XRF analysis

**Fig. 6.**
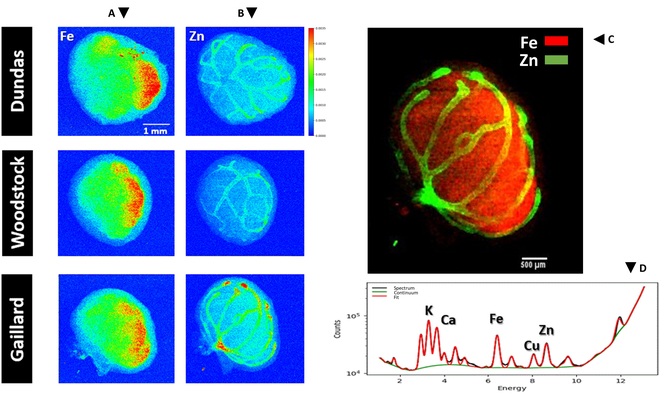
Distribution patterns of Fe (column A) and Zn (column B) in the root nodules of the 3 soybean genotypes with varying N_2_-fixation capacities. The elemental maps were obtained through synchrotron x-ray fluorescence (SR-XRF) imaging, at a resolution of 20 μm, following spectral deconvolution by PyMca. The overlay SR-XRF image of Fe (red) and Zn (green) in panel (C) reveals the distinct and predominant localization of Fe within the bacteroid containing cells of the central infected zone (red) tissue, and Zn within the nodule vasculature (green) in a soybean nodule. In this image, the color intensities for Fe and Zn were adjusted to reveal the differential localization of the 2 elements. A representative x-ray fluorescence emission spectrum of a nodule is shown in the plot (D) (black line). The incident x-ray energy was 15 keV. The overall fit (red line) represents deconvoluted elemental peaks corresponding to the K shell emission lines, which are the most intense fluorescence lines emitted by the elements present in the nodule. The peaks of several elements along with Fe and Zn are labeled as references. The green line represents the background fit. The color bar next to the top right of the Fe and Zn images in the 3 soybean genotypes depicts the normalized XRF counts, with red and blue indicating the relative abundance of Fe and Zn within the nodules, ranging from high (red) to low (blue). The scale is consistent across all Fe and Zn images.

Representative distribution patterns of Fe and Zn in the intact, hydrated root nodules of the 3 soybean genotypes with varying N_2_-fixation capacities, obtained using SR-XRF imaging at a spatial resolution of 20 μm, are presented in Fig. 6. The primary assessments of N_2_-fixation efficiencies of these genotypes using the ^15^N natural abundance method showed the significant superiority of Dundas in N_2_-fixation in comparison to Gaillard, while Woodstock showed an intermediate efficiency in N_2_-fixation. In terms of root system size, Woodstock and Gaillard exhibited the largest and smallest root system, respectively (Tables S2 and S3). In all 3 examined genotypes, the SR-XRF imaging of nodules revealed a distinct and predominant localization of Fe within CIZ and Zn within VB tissues. Notably, the XRF map of Zn and its localization within nodule VBs was quite specific for Zn compared with other essential metals in the nodule (e.g., Co, Ni, and Cu) that were analyzed and mapped (maps of other elements not shown).

**Fig. 7.**
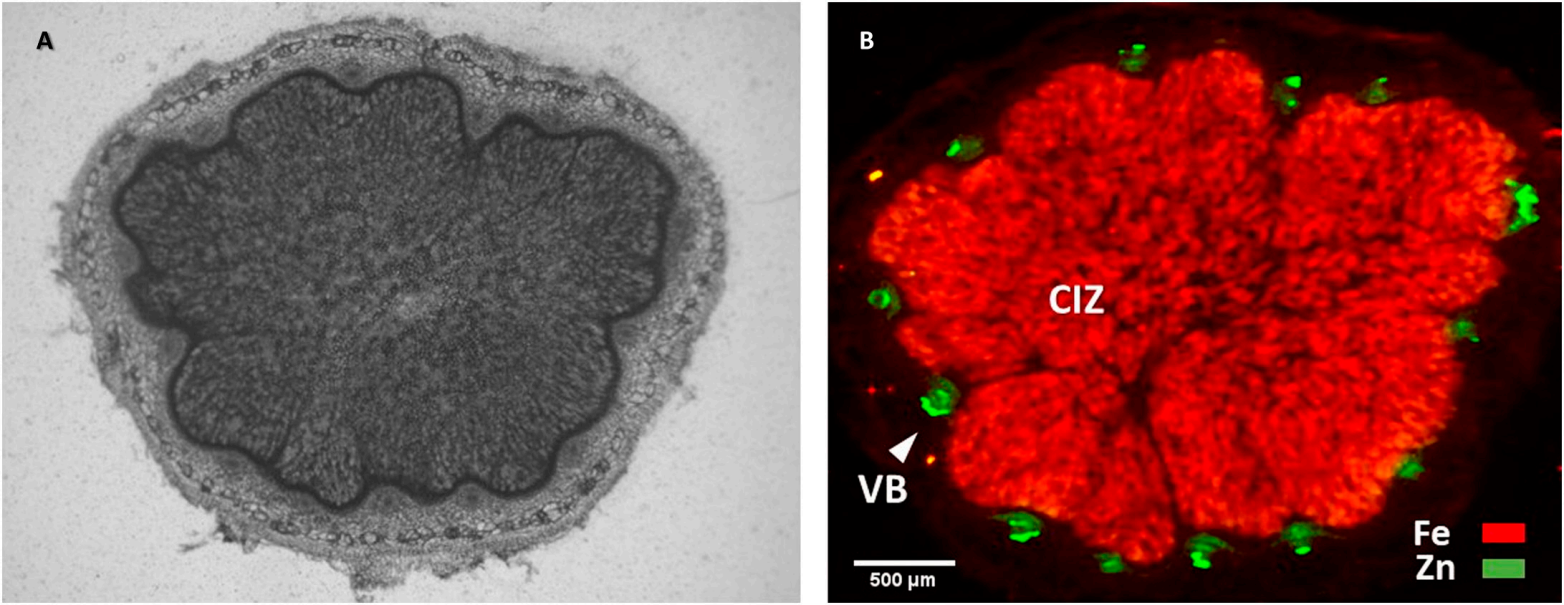
Mapping of Fe and Zn within a root nodule section using synchrotron XRF imaging at 5 μm resolution with a dwell time of 100 ms. The 100-μm-thick section was obtained from a fresh soybean nodule of a 4-week-old soybean plant (cv. Woodstock) (A). Note the distinct localization of Zn (green) within the nodule vascular tissues (VB) and Fe (red) within the central infected zone (CIZ) (B).

This differential localization of Fe and Zn enabled 2D visualization of nodular CIZ and VB tissues, as seen in the overlay of Fe (red color) and Zn (green color) spatial localization in the SR-XRF nodule image in the top-right panel of Fig. 6. The consistency of the distinct localization of Fe and Zn in internal tissues of root nodules of various soybean genotypes revealed the utility of the SR-XRF imaging technique for visualizing internal tissues of CIZ and VB in nodules through mapping of these elements in soybean root nodules. However, due to the x-ray beam’s ability to penetrate the entire nodule volume, fluorescence is emitted from the entire volume along the path of the beam [46]. Consequently, the 2D XRF images represent a compressed representation of the Fe or Zn, present within the nodule volume, in a single plane. To better confirm the differential localization of Fe and Zn within the nodule CIZ and VB tissues, the distribution of these elements was mapped within the 100-μm-thick nodule sections of the 3 soybean genotypes using SR-XRF imaging at a higher resolution of 5 μm (Fig. 7). The distribution patterns of Fe and Zn within the nodule sections of the 3 soybean genotypes closely resembled the distribution patterns observed in their intact nodules. Figure 7B shows a representative overlay SR-XRF image of Fe and Zn within the nodule section of cv. Woodstock.

These results suggest that due to the distinctive localization of Fe within CIZ tissue and Zn within VB tissue in soybean root nodules, SR-XRF imaging can be employed for non-invasive visualization of these internal structures of soybean nodules in 2 dimensions. To our knowledge, this is the first report on visualizing internal tissues of CIZ and VBs in soybean root nodules using the SR-XRF technique. However, care must be taken using 2D elemental maps of intact nodules for quantitative comparisons, whether among the Fe and Zn maps of the same genotype or between different genotypes for a specific element (Fig. 6). This caution is necessary due to the different quantum yields of Fe and Zn, as well as irregularities in nodules’ thickness leading to the variations in self-absorption of emitted XRF photons within the nodule. These factors can introduce discrepancies in the observed intensities in the image maps, potentially leading to inaccuracies in estimating concentration differences.

SR-XRF tomography has proven its utility in visualizing the localization of specific metals in seeds, as demonstrated by van der Ent et al. [47] for Ni-Cd-Zn accumulation in seeds of the Zn/Cd hyperaccumulator, *Noccaea caerulescens*, and Kim et al. [48] for Fe localization in Arabidopsis seeds. Employing this technique to achieve 3D visualization of the internal nodule structures of CIZ and VBs through 2D XRF mapping of Fe and Zn within intact root nodules holds promise. However, the longer acquisition times required for scans from multiple angles may necessitate the use of faster and more efficient fluorescence detectors to prevent beam-induced damage to the hydrated nodules during the prolonged scans [46,49].

**Fig. 8.**
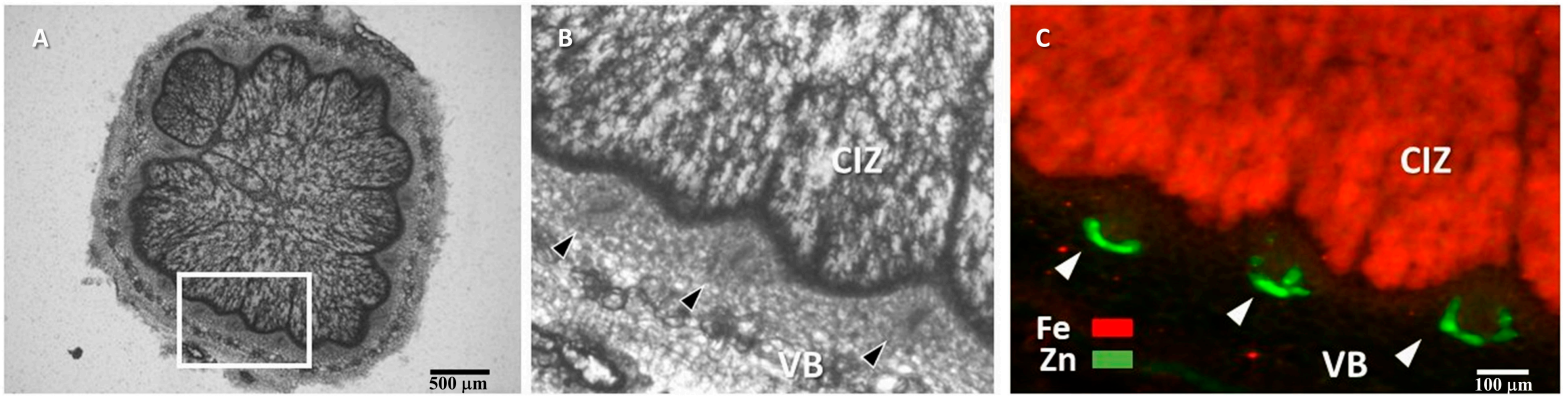
Fine mapping of Fe and Zn distributions within the root nodule VBs using synchrotron XRF imaging at a spatial resolution of 2 μm and a dwell time of 100 ms. The 100-μm-thick section was obtained from a fresh soybean nodule of a 4-week-old soybean plant (cv. Dundas). The marked area in image (A) shows the region of interest (ROI) containing 3 VBs. In (B), this ROI was selected for high-resolution scanning. The synchrotron XRF image (C) was then acquired from this selected ROI (B). To locate the position of VBs in the nodule section, the entire section first was initially scanned at a low spatial resolution of 30 μm with a short dwell time of 20 ms. Once the ROI was determined in the nodule section, the ROI was scanned at a resolution of 2 μm, with an exposure time of 100 ms. The circular pattern of Zn (green) localization within the VBs in image (C) corresponds to the arrangement of the vascular endodermis surrounding the nodule VBs (cf. Fig. 7B). The arrowheads in image (B) and (C) indicate the nodule VBs surrounding the central infected zone (CIZ), enriched in Fe (red).

To investigate the localization of Zn in specific cell types within the nodule VBs, a region was identified within a nodule section of cv. Dundas where 3 VBs were present (Fig. 8). The VBs were then scanned at a high spatial resolution of 2 μm. The fine mapping of Zn revealed its prominent localization within the nodule VBs, mainly where vascular endodermis cells are present (Fig. 8).

Due to its role as a micronutrient, Zn is essential for plant growth. However, when present in excessive amounts, Zn can become toxic. To prevent toxicity and maintain Zn levels within the non-toxic but Zn essential range in the plant shoots, plants have developed an adaptive mechanism that involves sequestering excess Zn within plant root endodermal cells [29,31,32]. The Casparian strip, an impermeable diffusion barrier made of suberin and lignin deposited in the cell wall around the endodermal cells, effectively blocks the apoplastic pathway for solute movement in the apoplast from the root cortex into the stele. As a result, the transport of Zn from the root cortex to the xylem takes place solely through the symplastic pathway, necessitating the involvement of active endodermal plasma membrane Zn transporters and also cell-to-cell symplastic plasmodesmal connections between endodermal cells and cells on each side of the endodermis. Lu et al. [33] conducted a study using μ-XRF mapping to investigate the distribution patterns of Zn in the roots of Zn hyperaccumulating (HP) and non-hyperaccumulating (NHP) ecotypes of *Sedum alfredii*. The study revealed a prominent localization of Zn within the root stele of NHP plants, as determined by the analysis of μ-XRF images. Furthermore, the concentration of Zn measured in the xylem sap of NHP plants was significantly lower than that in HP plant roots. These results suggest that the NHP ecotype employs a strategy of sequestering Zn within the tissues surrounding the root vasculature, thereby limiting its availability for xylem loading. In contrast, the hyperaccumulation of Zn in the shoot of the HP ecotype is largely attributed to the efficient loading of Zn into the xylem in HP roots, which relies on active membrane Zn transporters mediating Zn efflux from the xylem parenchyma into the xylem vessels, with subsequent storage of high Zn levels in leaves, via sequestration in leaf vacuoles and employment of Zn chelating compounds [33,50].

Research suggests that Zn is delivered to the rhizobia-infected cells through the nodule vasculature [51,52], in a process that resembles metal delivery to plant shoots [53]. To the authors’ knowledge, no prior studies have reported prominent localization of Zn within the nodule vascular endodermis. It remains unknown whether this typical localization of Zn within the nodule vascular endodermis follows a similar adaptive strategy, reported for the sequestration of Zn within the plant root stele [31,32], in order to prevent Zn toxicity in the inner nodule tissues and, likely, to bacteroids. Furthermore, Fe and Zn have a chemical similarity in their divalent cationic forms (as Fe can be Fe^3+^ or Fe^2+^ and in the low oxygen environment of the CIZ, it is possible that Fe^2+^ predominates) and shares some metal transporters with Zn^2+^, resulting in mutual interference in their uptake, transport, and distribution within plant tissues [54,55]. Recently, Castro-Rodríguez et al. [53] identified the MtYSL3 transporter expressed in the plasma membrane of endodermal cells in nodule vasculature in model legume, *Medicago truncatula*, that is involved in both Zn and Fe delivery to nodules. Therefore, it is plausible that the high demand for Fe in the CIZ tissue (cf. Figs. 7 and 8) could hinder the transport of Zn out of the nodule vasculature.

**Fig. 9.**
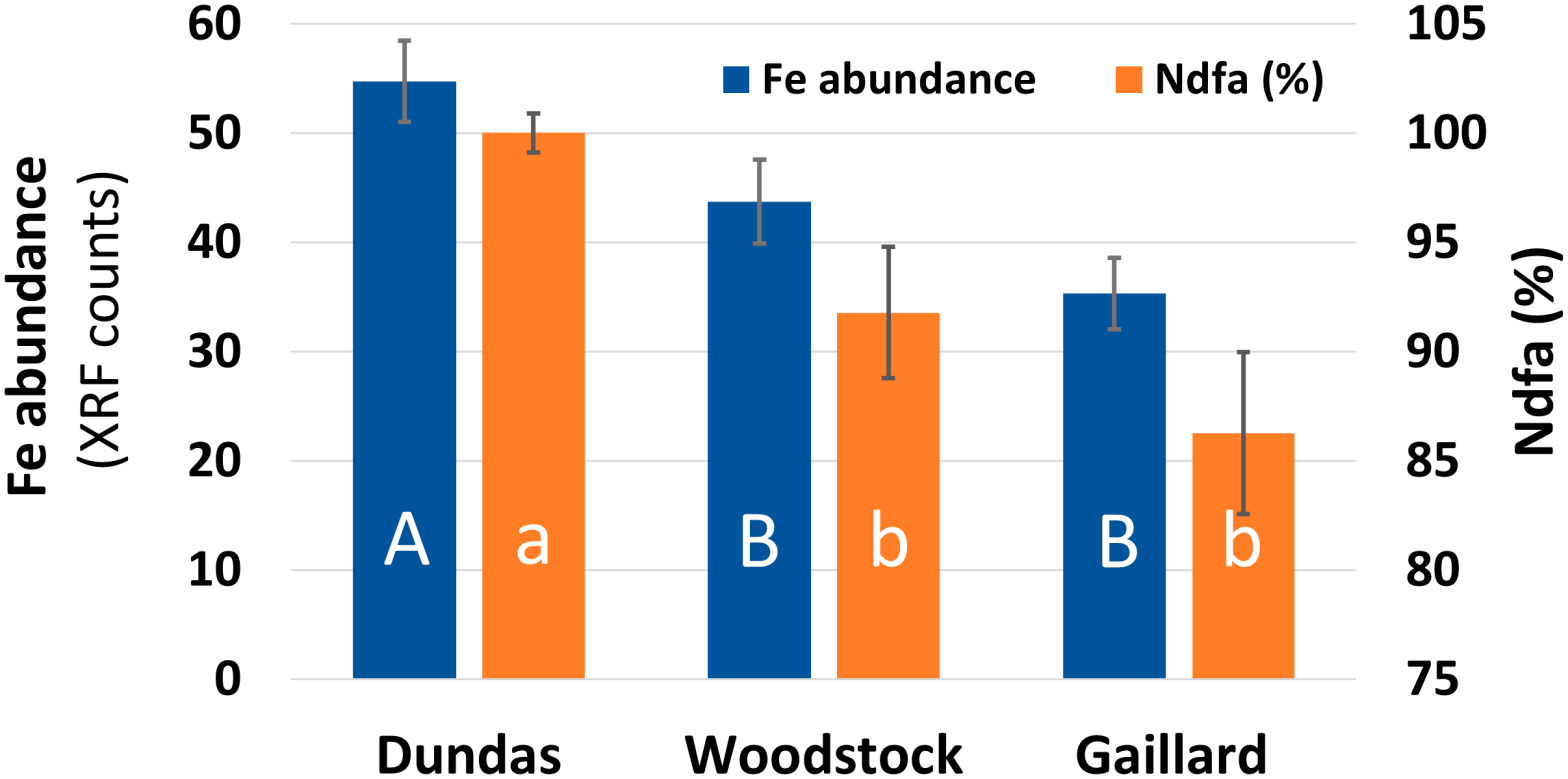
Relationship between root nodule Fe abundance and N_2_-fixation efficiency (%Ndfa) in the 3 soybean genotypes. The abundance of Fe in root nodules was determined by calculating the total normalized XRF counts under the Fe peak (K shell line) in XRF spectra obtained through the SR-XRF imaging of their intact root nodules. The N_2_-fixation capacities of the soybean genotypes were assessed using the ^15^N natural abundance method and expressed as %N derived from air (%Ndfa, cf. Table S1’s caption for additional information on the methodology). The %Ndfa results were obtained through combined analysis of 2 datasets obtained from separate phenotyping events, as presented in Tables S2 and S3. Data points and error bars represent means and standard errors of 15 and 10 replicates for Fe abundance and %Ndfa, respectively. Uppercase and lowercase letters indicate the significance of differences between genotypes for Fe abundance and %Ndfa, respectively, at a significance level of *P* < 0.05.

Semi-quantitative analysis of the SR-XRF data showed that the differences observed in root nodule Fe fluorescence between the 3 soybean genotypes was consistent with their differences in N_2_-fixation as measured by the ^15^N natural abundance method (Fig. 9). Research have shown that root nodules are a large sink for Fe accumulation. This is because CIZ is the site of N_2_-fixation where multiple Fe-containing enzymes and proteins involving in N_2_-fixation such as nitrogenase, leghemoglobin, and ferredoxin are highly abundant [26]. These results suggest that SR-XRF imaging can be employed for in situ assessment of nodule activity. However, we note that the abundance of the Fe within the nodules was not corrected for variations in nodule thickness across their geometry. Therefore, any conclusion based on these quantitative results should be drawn with caution. However, to obtain a more accurate quantification of elements within intact biological samples, such as plant materials, which often exhibit irregular thickness across their geometry, alternative methods have been proposed. These methods involve measuring the transmittance of the sample and assuming that the absorption characteristics of the plant material can be approximated as water [56].

## Conclusion

Non-destructive 3D visualization and volume quantification of the internal root nodule tissues of CIZ and VBs are essential to assess their functional importance in N_2_-fixation in the legume–rhizobia association. This study, for the first time, introduced successful application of synchrotron x-ray microtomography techniques for non-invasive 3D visualization and quantification of these functionally important root nodule structures. This was achieved through rapid imaging of fresh root nodules without the need for labor-intensive sample preparation or the use of contrast-enhancing agents. The high resolution of μ-CT images enabled easy differentiation of CIZ and VB structures within the root nodule tomograms. The 3D reconstruction of CIZ and VB structures was facilitated by employing Biomedisa’s smart interpolation algorithm, which enables rapid and accurate (semi)automated segmentation of these nodular tissues based on the sparsely pre-segmented slices within the volume image. This, in turn, markedly speeded up the subsequent process of volumetric quantification of nodular CIZ and VB structures using Biomedisa’s 3D models. The results obtained through our experiments revealed notable variation in the volume of VBs among examined soybean genotypes with varying N_2_-fixation capacities. In future work, it would be valuable to explore the potential of utilizing deep neural networks in Biomedisa for automatic segmentation of these nodule structures. Such an approach has the potential to speed up quantitative analysis, offering opportunities for further advancements in this field. Additionally, employing SR-XRF imaging in this study revealed the distinct localization of Fe within CIZ and Zn in VBs tissues, showcasing the practical benefit of SR-XRF imaging of nodules for visualizing the CIZ and VB tissues in 2D through mapping of Fe and Zn in nodule. However, future studies could also explore the potential of SR-XRF tomography for 3D visualization of nodule functional structures through XRF mapping of Fe and Zn within intact nodules. Employing high-resolution SR-XRF imaging for the fine mapping of Zn in root nodule sections, this study provides the first evidence of the prominent sequestration of Zn within the vascular endodermal layer of VBs in soybean root nodules. The employed techniques allow for simultaneous imaging of multiple root nodules, which enhances the applicability of these methods for high-throughput phenotyping of functionally important structures of root nodules. The SR-μCT, as demonstrated here, can be implemented as a rapid, non-invasive tool to unravel the functional importance of root nodule CIZ and VB tissues in N_2_-fixation in symbiotic legume–rhizobia systems and to investigate the possible exploitation of these root nodule features as novel phenotypic traits in breeding for improved N_2_-fixation efficiency via the development of soybean cultivars with increased root nodule activity.

## Data Availability

All the data related to this paper are included in the paper and/or the Supplementary Materials.

## Supplementary Materials

Supplementary 1 Tables S1 to S3 Figs. S1 and S2 Movies S1 to S3 Reference [57]
