# Supplementary material for: Visualization and Quantitative Evaluation of Functional Structures of Soybean Root Nodules via Synchrotron X-ray Imaging
Source: Plant Phenomics. 2024 Jul 17;6:0203. doi: 10.34133/plantphenomics.0203 (PMC11254386; doi:10.34133/plantphenomics.0203)
Supplement: Supplementary 1 — Tables S1 to S3 Figs. S1 and S2 Movies S1 to S3 Reference [57] [file plantphenomics.0203.f1.zip › Supp Tables S1-S3, Figure Legends S1-S2, Video Legends S1-S3.pdf]

Title: Visualization and quantitative evaluation of functional structures of soybean root nodules via synchrotron X-ray imaging

Alireza Nakhforoosh, Emil Hallin, Chithra Karunakaran, Malgorzata Korbas, Jarvis Stobbs, Leon Kochian*

*Address correspondence to: leon.kochian@usask.ca

Supplementary Tables (Table S1-S3)

Table S1. Mean values and analysis of variance for shoot, root and nodule traits associated with N_2-fixation in three soybean genotypes used for the experiments at the BMIT-BM beamline at the Canadian Light Source (CLS) [a]

Genotype	Dry weight: Shoot (g/pl)	Dry weight: Root (g/pl)	Root nodules: Fresh weight (g/pl)	Root nodules: Number (#)	Leaf Nitrogen [b] Concentration (mg/g)	$\delta^{15}N_{fix}$ [c] (‰)	Ndfa [d] (%)
PI209332	0.5	0.21	0.25	54.8	14.6	0.606	66.6
PI567651	0.7	0.26	0.34	90.5	17.4	0.004	94.3
Williams 82	1.1	0.39	0.56	129.7	15.0	-0.121	100.0
Mean	0.8	0.29	0.39	93.8	15.7	0.180	86.2
LSD (0.05)	0.180	0.068	0.086	39.26	2.68	0.563	25.91
P value	<.001	<.001	<.0001	<.001	0.075	<.05	<.05

[a] Phenotyping of soybean genotypes was conducted on five 4-week-old soybean plants under controlled conditions as mentioned in the text. The mean values are the average of six replications.

[b] The nitrogen content in the plant material was assessed by analyzing a 0.25-gram dried subsample obtained from all collected plant leaves.

[c] ^{15}N natural abundance ($\delta^{15}N_{fix}$) was performed on plant leaves. All plant leaves were collected, oven-dried at 60°C for 48 hours, and ground to a very fine powder using a mortar and pestle. A subsample of 3 mg was taken for the ^{15}N isotope analysis using a Costech ECS4010 elemental analyzer (Costech Analytical Technologies Inc. in Valencia, California) coupled to a Delta V mass spectrometer with Conflo IV interface (Thermo Scientific in Bremen, Germany).

[d] %N derived from air ($\%Ndfa=100\times[\delta^{15}N_{ref} - \delta^{15}N_{fix}]/[\delta^{15}N_{ref} - B]$) was estimated using the method described by Unkovich and Pate [36]. To calculate %Ndfa, the abundance of ^{15}N in non-fixing reference plant was estimated by measuring the abundance of ^{15}N in non-nodulated plants of the Dundas genotype that were watered with a (+N) nutrient solution ($\delta^{15}N_{ref}$= 2.0525‰). The B value was approximated using the average $\delta^{15}N_{fix}$ value of the genotype with the smallest $\delta^{15}N_{fix}$ value in each phenotyping experiment; here Williams 82 (B= -0.121‰).

Identification of high and low N_2-fixing soybean genotypes for experiments at the BioXAS-Imaging beamline at the Canadian Light Source

For the experiments at the BioXAS-Imaging beamline, we used high and low N_2-fixing soybean genotypes which were selected through the phenotyping of a set of 25 Canadian short-season soybean genotypes for traits associated with N_2 fixation. The 25 soybean genotypes were chosen from a core set of 137 Canadian short-season soybean which had been previously phenotyped for traits related to root system architecture in rhizoboxes by Seck et al. [57]. In that study, soybean genotypes in the panel were clustered into seven clusters through a GWAS of the soybean association panel genotyped with 2.18 million SNPs via a combination of genotyping-by-sequencing and whole-genome sequencing. To ensure that our panel adequately represents the diversity of root traits observed in the original panel, we selected soybean genotypes from every cluster, with the number of selected genotypes from each cluster being proportional to the cluster's size.

Phenotyping of the 25 soybean genotypes was conducted in two separate cycles since 15 genotypes didn't germinate during the first cycle. These remaining 15 genotypes were phenotyped in the second round along with three checks (Dundas, Woodstock, and Gaillard) chosen from the first phenotyping event. The analysis of variances was performed separately for each phenotyping cycle, as shown in Tables S2 and S3.

Table S2. Mean values and analysis of variance for shoot, root and nodule traits associated with N_2 fixation in 10 soybean lines (1st screening cycle) from a pool of 25 representative lines selected from the Canadian short season GWAS panel (Seck et al [57]) to select high and low N_2-fixing soybean genotypes for the experiment at the BioXAS-Imaging beamline at the CLS [a]

Genotype	Dry weight: Shoot (g/pl)	Dry weight: Root (g/pl)	Dry weight: Root nodules (g/pl)	Number of nodules (#)	Nitrogen [b] (mg/g)	$\delta^{15}N_{fix}$ (‰)	Ndfa [b] (%)
9063	2.9	0.64	0.065	83.0	30.5	-1.137	86.9
Dundas	2.3	0.66	0.085	127.2	24.5	-1.618	100.0
Evans	2.9	0.86	0.083	109.0	20.7	-1.130	86.8
Gaillard	2.4	0.41	0.071	99.8	32.6	-0.758	76.6
KG-41	2.5	0.46	0.073	124.3	35.7	-1.355	92.9
OAC Ayton	1.9	0.70	0.070	119.7	37.5	-0.987	82.8
OAC Lakeview	2.7	0.55	0.099	137.8	32.6	-1.286	91.0
PRO25-53	3.6	0.83	0.107	127.5	23.6	-1.338	92.4
SECAN 8_24	2.1	0.57	0.052	88.0	34.1	-0.778	77.1
Woodstock	3.4	1.01	0.126	183.8	24.9	-1.104	86.0
Mean	2.7	0.66	0.084	122.0	29.7	-1.150	87.2
LSD (0.05)	0.51	0.154	0.0277	37.82	4.78	0.323	8.81
P value	<.0001	<.0001	<.0001	<.001	<.0001	<.0001	<.0001

[a] see the caption of Table S1 for the phenotyping conditions of the soybean genotypes. [b] The B value was approximated using the average $\delta^{15}N_{fix}$ value of the Dundas (-1.618‰). The mean values are averaged over five replications.

Table S3. Mean values and analysis of variance for shoot, root and nodule traits associated with N_2 fixation in 15 soybean lines (2nd screening cycle) from a pool of 25 representative lines from the Canadian short season GWAS panel (Seck et al [57]) to select high and low N_2-fixing soybean genotypes for the experiment at the BioXAS-Imaging beamline at the CLS [a]

	Dry weight (g/pl)			Number of nodules	Nitrogen	$\delta^{15}N_{fix}$	Ndfa [b]
**Genotype**	Shoot	Root	Root nodules	(#)	(mg/g)	(‰)	(%)
40426Bp	2.8	1.01	0.143	148.4	34.2	-2.076	99.7
4067P17j	3.4	0.88	0.124	139.8	33.5	-1.824	93.6
Altesse	3.1	0.93	0.177	224.8	41.6	-1.750	91.8
Amasa	2.9	0.88	0.132	181.6	35.9	-1.788	92.7
DH420	3.9	1.01	0.179	227.7	30.6	-1.827	93.7
Dundas-check	2.7	0.95	0.141	176.8	31.6	-2.092	100.0
Gaillard-check	3.1	0.74	0.126	83.6	32.5	-1.922	95.9
Jutra	3.1	1.11	0.144	140.6	34.2	-2.030	98.6
Kamichis	3.5	1.03	0.172	160.4	29.9	-1.844	94.0
OAC 7-26C	3.5	1.06	0.190	177.6	34.4	-1.964	97.0
OAC 8-21C	2.9	1.12	0.159	151.8	34.2	-1.828	93.7
OAC Bayfield	2.9	0.89	0.162	152.0	37.0	-1.796	92.9
OT 11-09	3.8	1.08	0.146	132.8	29.3	-1.728	91.3
Roland	3.5	1.04	0.155	167.4	28.5	-2.066	99.4
S12-A5	2.7	0.93	0.157	116.6	35.9	-1.790	92.8
SECAN 9-38	2.8	1.07	0.153	164.2	34.6	-1.770	92.3
Walton	3.5	1.16	0.191	165.6	31.6	-1.876	94.9
Woodstock-	3.0	1.17	0.169	160.3	31.9	-2.050	99.0
Mean	3.2	1.00	0.155	158.0	33.5	-1.890	95.2
LSD (0.05)	0.44	0.151	0.0332	37.12	2.96	0.106	2.56
P value	<.0001	<.0001	<.001	<.0001	<.0001	<.0001	<.0001

[a] see the caption of Table S1 for the phenotyping conditions of the soybean genotypes. [b] The B value was approximated using the average $\delta^{15}N_{fix}$ value of Dundas (-2.092‰). The mean values are averaged over five replications.

Supplementary Figure Legends (Fig. S1-S2)

Fig. S1. The flowchart illustrates the workflow for 3D visualization and volume quantification of functional structures of soybean root nodules using synchrotron X-ray μ-CT (SR- μCT) and the Biomedisa segmentation software. Tomographic scans of the root nodules were acquired at the BMIT-BM beamline at the Canadian Light Source (a-b). Following the μ-CT scans, 2D projection images were reconstructed, forming the original image stack (c). Manual segmentation of the nodular central infected zone (CIZ) and vascular bundle (VB) tissues were performed on every 100 (for CIZ) and 40th (for VB's)

slice within this stack to produce labeled image stacks (d). The original (c) and labeled (d) image stacks served as input data for (semi)automatic segmentation using Biomedisa software. Biomedisa's smart interpolation algorithm assigned labels to the features of interest within unlabeled slices, resulting in a 3D model of the entire structure of functional nodule tissues (e). The generated 3D models were visually checked in Avizo software for segmentation errors (f). In the case of identified gaps or discontinuities, manual labeling of additional slices within these regions was conducted, and the segmentation process was iterated until satisfactory results were achieved. Post-processing steps, including smoothing, hole filling, and removal of outliers, were then applied to the 3D models prior to volumetric measurements, all performed within Avizo software (g).

Fig S2. The workflow demonstrates 2D visualization of functional structures of soybean root nodules (a) using synchrotron the X-ray fluorescence SR-XRF imaging technique. SR-XRF imaging data were acquired at the BioXAS-Imaging beamline of the CLS. The nodular central infected zone (CIZ) and vascular bundle (VB) tissues were visualized in 2D through mapping Fe and Zn and by XRF imaging of fresh, intact root nodules in macro mode at a spatial resolution of 20 μm (b-c). The micro mode of the BioXAS-Imaging (with beam size of 5 μm and 2 μm) was employed to further examine the specific localization of Fe and Zn within nodular CIZ and VB tissues, respectively. This involved fine mapping of Fe and Zn distributions within 100-micron thick root nodule sections (d-f).

Supplementary video legends (Video S1-S3)

Video S1. Volume rendering of a soybean root nodule (genotype PI209332) obtained through synchrotron X-ray μCT scanning.

Video S2. Visualization of the vascular connectivity between plant root and nodule in a 3D rendered soybean root nodule (PI209332 genotype). The movie, in the left panel, shows sequential XY-plane slicing of a 3D reconstructed nodule along the Z-axis from top of the root nodule downward. The movie, in the right panel, displays the corresponding 2D slices. As the slicing approaches the junction of the root and nodule, the movies are slowed down to enhance visualization of the vascular interconnectivity between the root and nodule. The red arrow in the image indicates a large (7 μm wide), air-filled nodule vessel arising from the root vasculature. Due to the relatively lower X-ray attenuation resulting from the lack of water in the vessel, the specified embolized nodule vessel exhibits good contrast (black), making it highly visible compared to the water-filled vessels surrounding it (gray).

Video S3 Volume rendering of the overlay of 3D models of CIZ (blue) and VB (red) tissues projected into the volume-rendered soybean root nodule (genotype PI209332), obtained through synchrotron μCT imaging.
